# Gain-of-function mutations in the RodZ juxtamembrane domain promote Rod system activity in *Escherichia coli*

**DOI:** 10.64898/2026.10.11.758364

**Published:** 2026-10-12

**Authors:** Katherine A. Suarez, Thomas G. Bernhardt

## Abstract

The peptidoglycan (PG) cell wall protects bacteria from osmotic lysis and determines their shape. In rod-shaped bacteria, the lateral wall is elongated by the Rod system (elongasome). This synthetic machine is built around the RodA-PBP2 PG synthase complex, the actin-like protein MreB, and the accessory proteins MreC, MreD, and RodZ. Notably, RodZ shares a similar domain organization with FtsN, an essential division protein that triggers PG synthesis by the division apparatus (divisome). Both are single pass transmembrane proteins that bind actin-like proteins in the cytoplasm and have largely unstructured periplasmic regions capped by small, folded C-terminal domains. We therefore investigated whether RodZ might function like FtsN in activating the Rod system. To this end, gain-of-function variants of *Escherichia coli* RodZ were isolated that restored growth and shape to cells producing a hypomorphic *mreC* mutant. Production of these RodZ variants in wild-type cells increased resistance to the MreB antagonist A22 and enhanced sensitivity to the beta-lactam mecillinam. These phenotypes have all previously been associated with Rod system hyperactivation, and we found that they were caused by substitutions in the cytoplasmic juxtamembrane (JM) domain of RodZ. This domain is analogous to the N-terminus of FtsN known to promote divisome activation via its interaction with filaments of the actin-like FtsA protein. Structural modeling indicates that the JM domain of RodZ is similarly likely to associate with MreB filaments. Our results therefore support a model in which RodZ activates PG synthesis by the Rod system via a mechanism analogous to divisome activation by FtsN.

**IMPORTANCE:**

Cell elongation in rod-shaped bacteria like *Escherichia coli* is mediated by the Rod system, and cell division is promoted by the divisome apparatus. It has long been recognized that these two machineries employ related components such as the peptidoglycan synthases RodA-PBP2 in the Rod system and FtsW-FtsI (PBP3) in the divisome. Here, we provide evidence that the RodZ protein of the Rod system plays a role in the activation of PG synthesis by the elongation machinery analogous to that performed by FtsN in divisome activation. The results suggest that the elongation and division machineries not only share related components but are also activated by similar regulatory mechanisms.

